# The Impact of SARS-CoV-2 Infection on Fertility and Female and Male Reproductive Systems

**DOI:** 10.3390/jcm10194520

**Published:** 2021-09-29

**Authors:** Agnieszka Markiewicz-Gospodarek, Paulina Wdowiak, Marcin Czeczelewski, Alicja Forma, Jolanta Flieger, Jacek Januszewski, Elżbieta Radzikowska-Büchner, Jacek Baj

**Affiliations:** 1 Chair and Department of Anatomy, Medical University of Lublin, 20-090 Lublin, Poland; agnieszka.markiewicz-gospodarek@umlub.pl (A.M.-G.); paulina.wdowiak@umlub.pl (P.W.); marcin.czeczelewski@gmail.com (M.C.); 2 Chair and Department of Forensic Medicine, Medical University of Lublin, 20-090 Lublin, Poland; aforma@onet.pl; 3 Department of Analytical Chemistry, Medical University of Lublin, 20-093 Lublin, Poland; jolanta.flieger@umlub.pl; 4 Department of Plastic, Reconstructive and Maxillary Surgery, Central Clinical Hospital MSWiA, 02-507 Warsaw, Poland; jacek.januszewski000@gmail.com (J.J.); eradzikowska@radzikowskaclinic.pl (E.R.-B.)

**Keywords:** SARS-CoV2, COVID-19, pandemic, reproductive complications, male fertility, female fertility

## Abstract

The severe acute respiratory syndrome coronavirus 2 (SARS-CoV-2) infection remains a huge challenge for contemporary healthcare systems. Apart from widely reported acute respiratory distress syndrome (ARDS), the virus affects many other systems inducing a vast number of symptoms such as gastrointestinal, neurological, dermatological, cardiovascular, and many more. Currently it has also been hypothesized that the virus might affect female and male reproductive systems; SARS-CoV-2 infection could also have a role in potential disturbances to human fertility. In this article, we aimed to review the latest literature regarding the potential effects of SARS-CoV-2 infection on female and male reproductive systems as well as fertility, in general.

## 1. Introduction

At the end of December 2019, a new virus, officially recognized as the severe acute respiratory syndrome 2 (SARS-CoV2) coronavirus, emerged. In March 2020, the World Health Organization (WHO) announced a global pandemic related to the appearance of SARS-CoV-2. The SARS-CoV-2 virus mainly affects the respiratory system causing COVID-19 disease [1]. It causes cough, fever, difficulties in breathing such as shortness of breath, and severe conditions such as pneumonia [1,2]. It has been reported that the death rate from COVID-19 in various countries ranges from 1 to 4%, with an increased risk of its occurrence in the elderly and in patients with co-existent diseases. SARS-CoV-2 primarily spreads via the airborne route [3], however, other transmission routes have been hypothesized as well. The presence of SARS-CoV-2 in blood samples was confirmed by positive PCR test results [4,5,6]. The virus affects the target host cells by binding to the ACE2 (angiotensin-converting enzyme) molecule and modifies the expression of this receptor in host cells [7]. ACE2 is a member of the angiotensin-converting enzyme (ACE) family and converts angiotensin 1 into angiotensin 1–9 as well as angiotensin 2 into Ang 1−7 [8]. ACE2 receptors take part in physiological functions and are expressed in numerous organs including ovaries, uterus, vagina, and placenta [9]. Therefore, SARS-CoV-2 may have a potential influence on female reproductive organs [10]. Current data on the clinical features of SARS-CoV-2 also show that co-expression of ACE2 and transmembrane serine protease 2 (TMPRSS2) may facilitate virus entry [11,12]. TMPRSS2 is necessary to cleave the viral S protein and facilitate the fusion to the host cell [13]. This protease is broadly expressed in many human organs as compared to ACE2. Hence, ACE2 receptor may be primarily responsible for whether respective cells are vulnerable to SARS-CoV-2 infection [14]. It is crucial to investigate the potential effects of SARS-CoV-2 infection on female and male fertility, as so far no comprehensive evidence has been provided for such association.

## 2. Aim of the Review and Search Strategy

Major objective of this review was to investigate the potential role of SARS-CoV-2 infection on the functioning of female and male reproductive organs (with an emphasis on the accumulation of viral particles within the reproductive organs), any disruptions concerning female and male fertility, as well as hormonal dysfunctions or any disruptions regarding the pregnancy course. An analysis of the articles available on PubMed, Web of Science, Scopus, and Cochrane databases was performed by four independent researchers on 30 August 2021. The identification of the articles for final analysis included usage following key-words: (SARS-CoV-2 OR COVID-19) AND (reproductive organs OR sex hormones OR pregnancy OR fertility). The literature search included both studies performed on humans and animals; concerning language restrictions, the authors chose only those that were written in English. Finally, 95 articles were included in this narrative review.

## 3. SARS-CoV-2 in Female Reproductive Organs

The influence of SARS-CoV-2 infection on the genitourinary system remains unknown. Data from several papers indicate that the virus may be present in vaginal swabs [9]. Shwartz et al. analyzed both the vaginal and nasopharyngeal swabs obtained from 35 premenopausal and postmenopausal women, indicating that two of them had positive vaginal RT-PCR for SARS-CoV-2; [15]. Alexandre J. Vivanti presented a case of reinforcing the possibility of vertical transmission [16].

SARS-CoV-2 uses ACE2 as a receptor for entry into the host cell; ACE2 is widely present in the human ovaries as well as in other species such as rats [4,17]. Therefore, it was assumed that female reproductive organs might be highly vulnerable to SARS-CoV-2 entry. Gonadotropin facilitates the changes in the ovarian expression of ACE2 which is a crucial factor in controlling the malformation of the follicle [18]. Analysis of the RNA sequencing data allowed the expression of ACE2, TMPRSS2, the cysteine protease cathepsin (CTSL), and the receptor basigin (BSG) that may have influenced viral entry to be estimated. ACEs, CTSL, and BSG were detectable in most samples as compared to TMPRSS2 expression which was very low or undetectable [13]. The analysis of the scRNAseq shows the scarcity of the data related to the inner cortex of human ovaries [19]. Several samples were obtained from women undergoing fertility preservation procedures, but inner cortex lacks the germ cells. Oocytes from non-human primate ovarian tissue exhibit co-expression of ACE2 and TMPRSS2. Very low co-expression was found in the oocytes from primordial follicles. The level of co-expression seems to increase during the follicular development, as it was detected on more than a half of the antral follicles. These oocytes undergo atrophy or ovulation, so the risk of infection is relatively low. Also, the surrounding environment, the cumulus cells, lack the ACE2 receptors [13]. Such data cannot exclude the fact that SARS-CoV-2 might attack ovarian tissue and granulosa cells, thereupon the ovarian function may be abnormal and oocyte quality decreased. The downregulation of ACE2 receptor after the viral infection may cause variations in the follicular development and oocyte maturation [20]. In many fertility treatment centers, IVF procedures were postponed. Either a direct or indirect effect of SARS-CoV-2 on gametes and embryos cannot be excluded [21], either. The embryology laboratory and staff must respect the precautions taken during all procedures e.g., oocyte recovery [22].

The ACE2 RNA expression level was also determined in the human fallopian tube, endometrium, uterine cervix, and the vagina [23]; low expression levels were observed in all the tissues. Also, TMPRSS2 RNA has been noticed in all reproductive organs but it demonstrated significant higher expression in the male tissues [23,24]. It seems that SARS-CoV-2 is unlikely to infect ovaries or interrupt oogenesis [25]. Abhari et al. researched the number of ACE transcripts in the endometrium and the expression of the protein form. The expression of ACE2 RNA was low and no protein was noticed. TMPRSS4 was highly expressed during menstruation by cleaving S protein and enabling SARS-CoV-2 to bind to ACE2. The renin-angiotensin system is present in the uterus and this presence is limited to the endometrium (epithelial and stromal cells in particular). Other researchers indicate to a positive correlation between the ACE2 expression and age especially in the early secretory phase of the endometrium. Generally, the endometrium seems to present low susceptibility to SARS-CoV-2 infection because of low ACE2 and TMPRSS2 expression. Therefore, it is proposed that women’s age might constitute a risk factor in SARS-CoV-2 infection [26]. Other published studies on SARS-CoV-2 RNA presence in the vaginal fluid and cervical smears revealed that, respectively, 98.3% and 100% samples tested negatively [27]. 

It is crucial to estimate and predict whether and how SARS-CoV-2 targets and attacks the human reproductive system ultimately decreasing fertility. The available data and evidence provide no clear suggestion that male or female genital tracts might transfer the virus. Limited data hinder the estimation of SARS-CoV-2 virus influence on female reproductive organs. Considering the aforementioned information, it can be assumed that or knowledge regarding the possible risk of SARS-CoV-2 infection within the female reproductive organs is still highly limited. Thus, the outbreak of the COVID-19 pandemic imposes new conditions and significant challenges on the reproductive healthcare of the community. 

## 4. The Multi-Faceted Effects of COVID-19 on Women, Including Sex Hormones

Given the rapidly spreading coronavirus pandemic and its impact on the global healthcare system, innovative therapeutic strategies should be continually sought. It is estimated that the number of people who die from COVID-19 infection is lower in women compared with men [28]. So far, differences between men and women in response to induced inflammation have been documented (these differences can in part be attributed to sex steroid hormones) [29]. 

In previous studies in female mice, the protective role of estrogens in protecting against SARS-CoV infection was documented [10]. Estrogens have been hypothesized to be crucial in modulating viral infection and the progression of the disease via an action on immune/inflammatory responses and ACE2 expression [28]. Given the above evidence clearly demonstrating the main role of estrogen in the immune, and non-immune responses to viral infections, it is not surprising that postmenopausal women who developed COVID-19 had a more severe course of infection [30]. Additionally, ovariectomy or treatment of female mice with an estrogen receptor antagonist significantly increased the death rate, indicating a protective effect on estrogen receptor signaling in COVID-19. Overall, these data suggest that the sex differences in susceptibility to COVID-19 in mice are similar to those seen in humans, and indicate that estrogen receptor signaling is a critical protective factor [31].

In a study by Ding et al., patients infected with COVID-19 were hospitalized in hospital in Tongji. The patients were divided into three groups—a first group of non-menopausal women, a second group of menopausal women, and a third group of men. The men oscillated around the same age as the woman. In the above study, no significant relationships between men and women were found. On the other hand, there were significant differences between the non-menopausal women and men of the same age, both in terms of the disease severity and clinical symptoms. A less severe course of COVID-19 disease has been observed in women. These results suggest that non-menopausal women might develop protective factors guarding them against the disease severity [32]. 

In another study by Phelan et al. aimed at examining the potential effects of a pandemic, i.e., changes in lifestyle, increased stress related to the occurrence of a pandemic and insecure existence, the general population of women of reproductive age was examined in terms of the menstrual cycle, libido, and lifestyle changes during the pandemic [33]. This study included 1031 women who completed the questionnaire via a computer link. Some of them i.e., 35/3.4% were positive for COVID-19, some i.e., 63/6.1% had symptoms, but this was not confirmed by the test, while the greater part i.e., 870/83% did not have COVID-19. The mean age of the women included in the study 15–54 years; the mean BMI was 25.8 ± 5.5 kg/m^2^. Looking the effects of COVID-19 on menstrual disorders, 46% of women reported on an overall change in their menstrual cycle, 53% reported an increase in PMS (premenstrual symptoms), while only 7% reported an improvement in PMS. The duration of the cycle itself varied, and the duration of bleeding was the same regardless of the pandemic [33]. This large, anonymous, observational study described that a large proportion of women experienced reproductive complications from the COVID-19 pandemic. A minority of women in the population experienced improvements in their health during the pandemic.

Some scientists such as Li et al. took a step forward by associating COVID-19 infection with genes encoded on the X chromosome, which may indicate the fact of a reduced mortality in women [34]. To understand the X-linked pathomechanism, we need to start with the biological role of ACE2. Biological functions of ACE2 could be divided into two categories—peptidase-dependent and peptidase-independent [8]. The peptidase-independent function of ACE2 mainly refers to the mediation of coronavirus infection. The gene encoding ACE2 is located on the X chromosome, being presented in two copies in females [35]. According to Lyon’s theory, one of the two X chromosomes from the mother or from father is transcriptionally silenced [36]. This complex silencing process, translating into X chromosome inactivation is a fundamental basis for ensuring balanced gene expression between the sexes [35]. Sometimes some genes (15–30%) located on the short arm of the chromosome avoid inactivation [37]. This has to do with the fact that ACE2 maps in the p22.2 band and, therefore, can avoid gene inactivation. Hence, this phenomenon could explain the differences observed among sexes regarding the ACE2 expression [35]. Therefore, future research on ACE2 levels in the genomic context, copy number variations, X-inactivation, and existing comorbidities is essential. This will help us understand the sex differences in ACE2-related pathophysiology, and how they are fully related to the SARS-CoV-2 pandemic [38].

## 5. COVID-19 and Pregnancy 

Pregnancy is generally considered a high-risk condition with respect to infectious conditions, as immune changes in pregnancy can greatly increase a woman’s susceptibility to pathogens, and their potential complications [12]. Pregnancy establishes a unique immunological condition, enabling the protection of the fetus from maternal rejection, providing an adequate fetal development at the same time preventing against microorganisms [39]. So far, approximately 55 pregnant women have been infected with SARS-CoV-2, and no mortality among these individuals has been associated with the disease course [40]. A total number of 46 neonates have been so far reported as being infected by the SARS-CoV-2, but there is no evidence indicating the vertical transmission of the virus [40].

**Figure 1:**
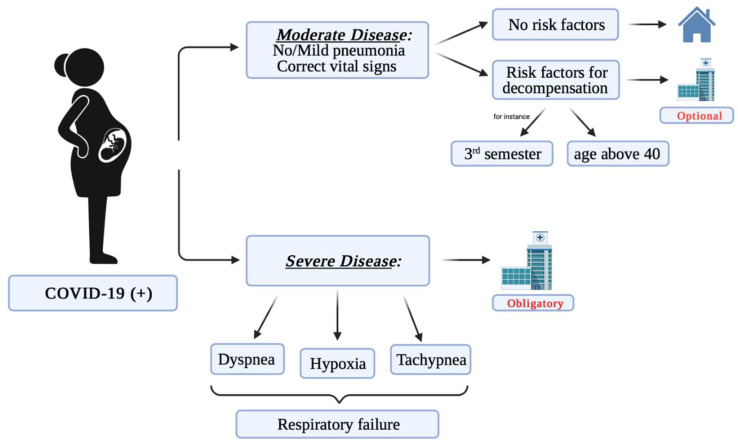
The potential course of COVID-19 depending on the severity of disease symptoms.

Scientists are trying to understand the impact of the COVID-19 pandemic on pregnant woman and the fetus. There are few confirmed cases in the literature related to women who tested positive for PCR during pregnancy (Figure 1). 

Preliminary reports from China suggested that pregnant women do not face an increased risk of complications related to SARS-CoV-2 in relation to the general population [9]. Further cases from the United States, for instance, provided contradictory information, suggesting that pregnant women may require mechanical ventilation and, in addition, are at a higher risk of death due to the occurrence of the disease [41]. In a study by Chen et al., nine women were diagnosed with COVID-19 infection in the third trimester of pregnancy [42]. In this relatively small group, the clinical presentation was similar to that observed in adult non-pregnant women i.e., fever (seven women), cough (four women), myalgia (three women), and sore throat accompanied by malaise (two women). Additionally, out of nine women, five had diagnosed lymphopenia. All of them suffered from pneumonia but required no additional intervention including mechanical ventilation and none of them died. All women had a cesarean delivery, and Apgar scores were 8–9 at 1 min and 9–10 at 5 min [43]. In another study by Breslin et al., no increased susceptibility of pregnant women to the risk of a severe course of the disease was found. Interestingly, most of pregnant women who were positive test for COVID-19 in the same study were asymptomatic [11].

In a recent review by Allotey et al. including 11,432 women, the most frequently reported symptoms were fever (40%) and cough (39%) [44]. Interestingly, pregnant women are more likely to remain asymptomatic as compared to non-pregnant women [45]. Additionally, pregnant women report muscle pain, diarrhea, headache, and sore throat less frequently [44]. In a review by Hong Liu et al., 18 pregnant women from the coronavirus pneumonia were considered. Their average age was 30 years of age. Each of these patients had one or two common clinical symptoms such as fever, cough, sore throat, diarrhea, and cholecystitis [46]. The birth weight (BW) of newborns from these mothers ranged from 1520–3820 g, and twins showed the lowest birth weight [47]. However, these women also had obstetric complications, such as pre-eclampsia, premature rupture of the mucosa, irregular contractions, and a history of stillbirth, indicating early intervention in the pregnancy [46]. Whether these complications were causally related to COVID-19, which in turn led to preterm labor, requires further investigation, although the characteristic immune response during pregnancy, which may result in a potential cytokine storm due to COVID-19 infection, needs to be considered. 

In the case of pregnant women who have been diagnosed with severe or critical COVID-19 infection, they are suspected to be at increased risk, and therefore more likely to be at risk of pregnancy loss, and preterm births. In studies conducted during hospitalization of pregnant women diagnosed with COVID-19, including 240 to 427 women, the risk of preterm labor ranged from 10 to 25%. At the same time, the percentage of preterm labor reached 60% in the women with a critical course of disease [48,49]. In an analysis of national surveillance data including pregnancy status of 409, 462 women with symptomatic COVID-19 illness, the adjusted risk ratio in pregnant women (vs. those of similar age and not pregnant) was 3.0 for intensive care unit admission, 2.9 for mechanical ventilation, and 1.7 for death [50]. Therefore, it is important to prevent critical COVID-19 infection for the mother and the fetus [49]. 

There are also studies that have examined the placenta of women infected with COVID-19. The study of three placentas delivered and taken from pregnant women with confirmed SARS-CoV-2 infection, infected in the third trimester of pregnancy, and performed with an unplanned cesarean section, describes various degrees of fibrin deposition [39]. In all samples taken from the aforementioned placenta were negative for the presence of SARS-CoV-2 nucleic acid [51]. In another study by Sahanes et al., which included 16 placentas taken from pregnant women with positive SACR-CoV-2 were examined, and the most significant finding is an increase in the rate of features of maternal vascular malperfusion (MVM), most prominently decidual arteriopathy including atherosis, fibrinoid necrosis, and mural hypertrophy of membrane arterioles [52]. No pathognomonic features were identified in the above study. In addition, there was also no increased rates of maternal vascular malperfusion, and intervillous thrombi, suggesting a common theme of abnormal maternal circulation, as well as an increased incidence of chorangiosis. The above results suggest that it is justified to increase the observation and supervision of women with a confirmed diagnosis of SARS-CoV-2 [52]. 

## 6. COVID-19 and Male Fertility

### 6.1. SARS-CoV-2 and Testes

The human testes are the male gonads which are composed of a highly integrated system of specialized cells ensuring a proper microenvironment to produce sperm cells. Each testicular lobule contains seminiferous tubules responsible for the process of spermatogenesis and steroid-secreting interstitial cells (Leydig) producing testosterone [53]. 

In seminiferous tubules, the maturing spermatocytes are separated from peripheral spermatogonia and spermatocytes by a blood–testis barrier formed by tight junctions between adjacent Sertoli cells. This barrier is crucial for the creation of an immune compartment that prevents spermatogenic cells against the body’s immune system [54]. Mature spermatids are released into the lumen of seminiferous tubules and pass into the epididymis for storage [55]. 

The capability of endocytosis of SARS-CoV-2 virus into target cells depends on the connection of viral S protein with the ACE2 receptor [7]. In 2004, Doughlas et al., reported the expression of ACE2 in adult Leydig and Sertoli cells of the human testis [56]. Wang et al. examined the ACE2 expression levels across testicular cell types. Sertoli and Leydig cells presented the highest expression followed by the spermatogonia [57]. Moreover, the ACE2 expression in the testes is age-dependent and peaks in men in their 30s [58].

Nevertheless, for ACE2-mediated SARS-CoV-2 endocytosis viral spike proteins must be primed by the transmembrane protease, serine 2 (TMPRSS2) [7,59]. The mRNA levels of TMPRSS2 protein in testes have been reported to be high in spermatogonia and spermatids [59]. Although both ACE2 and TMPRSS2 are expressed in testicular cells, high co-expression of the two proteins is not observed [13]. Pan and colleagues in the cohort study analyzed 34 adult males, and simultaneous expression of both proteins was rarely displayed in testicular cells according to the RNA profiling method [60].

Other potential cell receptors and cofactors of SARS-CoV-2 endocytosis are also present in testicular cells. CD147 and cathepsin L are mainly expressed in spermatocytes; cathepsin B in testicular macrophages [58]; exact distribution of CD26 and neuropilin-1 in testes needs to be established. The presence of ACE2 and other potential receptors in the testicular cells implies a possible vulnerability of testes to SARS-CoV-2 infection. However, the low co-expression of ACE2 and cofactors minimalizes the probability of direct invasion of the testes. 

Several histopathological studies directly investigated the presence of SARS-CoV-2 in the testicular tissue. Song et al. reported that the virus was not present in testes samples from a patient who died in the acute phase of COVID-19 [61]. However, in four other studies, the samples positive for virus particles were identified. Ma et al. found SARS-CoV-2 nucleic acid in two out of five testis samples through RT-PCR. It was further confirmed by immunohistochemistry using an anti-SARS-CoV spike S1 antibody which suggests that SARS-CoV-2 infects testicular cells through the spike glycoprotein binding mechanism. Final TEM analyses revealed coronavirus-like particles in the interstitial compartment of the testes of COVID-19 patients providing evidence that SARS-CoV-2 enters human testicular tissues [62]. A study by Bian et al. also detected the virus in one autopsy case of testis tissue via RT-PCR, TEM, and immunohistochemistry [63]. Yang et al. performed an autopsy of the testes of 12 COVID-19 patients. SARS-CoV-2 was detected in one patient who had a high viral load via RT-PCR; however, TEM failed to identify the virus [64]. In the study conducted by Achua et al. TEM revealed SARS-CoV-2 in the testis tissue of one of six COVID-19 patient autopsies [65]. However, whether the virus can enter the testes does not entirely depend on the viral mechanisms. A vital role may be played by imperfect blood-testes barrier, local inflammation, and high viremia. Scrotal heat stress, as such during viral infections, may cause leakage of the blood–testis barrier and passage of the virus to the testes [66,67]. 

However, it has been observed that direct virus access is not required to damage the male reproductive system. SARS-CoV-1 infection can cause severe orchitis with damaged germ cells, reduced mature spermatozoa, thickened base membranes, and significant leukocyte infiltration. The histopathological changes arise with the absence of SARS-CoV-1 in the testicular tissue, which implies that damage is caused by inflammation rather than viral infection [68]. Similar changes have been observed in COVID-19 patients. These included interstitial edema, congestion, inflammatory cell infiltrate, and red blood cell exudation in testes and epididymis, as well as thinning of seminiferous tubules [64,69,70]. 

Men with severe COVID-19 course presented a significantly higher possibility of orchitis than the non-severe COVID-19 patients [71]. Patients with severe COVID-19 infection had high levels of inflammatory cytokines: IL-2, IL-6, IL-7, IL-10, TNF-α, and MCP-1 [71,72]. High blood plasma concentration of these cytokines may lead to damage of the blood-testes barrier and induce inflammation in seminiferous tubules [66,73,74]. Moreover, inflammation-induced blood–testis barrier damage could be a potential explanation for the presence of SARS-CoV-2 in the testes of patients with a severe form of the disease [63].

### 6.2. SARS-CoV-2 and Semen

Li et al. identified SARS-CoV-2 RNA in the semen samples of four patients with acute infection and two that had recovered from the disease. The interval times from onset of symptoms to the testing of semen were 6–11 days and 12–16, respectively [75]. 

However other studies have identified no virus in the semen of convalescent men or during acute infection [76,77,78,79,80,81,82,83,84]. Most of these studies collected semen samples in a longer time interval from the onset of symptoms, which may suggest that SARS-CoV-2 penetrates to the semen at an earlier stage of the disease. Nonetheless, a study by Kayaaslan et al. collected the semen within 0–7 days and still reported negative viral RNA [85]. Therefore, the reasons for false-positive results of SARS-CoV-2 in semen might be considered. These include unknown conditions of sperm collection and detection limits of RT-PCR; imperfect specificity of commercial kits available for the detection of SARS-CoV-2 [86] and possible urine origin of viral RNA [87]. 

Apart from the presence or absence of a virus in semen, COVID-19 may impact spermatogenesis. The reported changes in semen include poor sperm morphology, decreased concentration and motility as well as increased sperm DNA fragmentation [69,77,78,79,83,84]. Additionally, immune factors, including IL6, TNF-α, MCP-1, were increased in the semen [69]. A decline in sperm quality has been found in patients with a moderate course of the disease which may be associated with fever and inflammation [77]. Fever is one of the most common COVID-19 symptoms [72] and harms spermatogenesis and impoverishes sperm quality [88]. However, high temperature usually does not result in irreversible damage to male fertility. The return to the basal state of the sperm parameters can take up to 3 months [89]. It should be emphasized that increased sperm DNA fragmentation leads to decreased fertility, poor embryo quality, and increased embryonic loss [90,91]. Therefore, it has been recommended to monitor sperm parameters and to delay ART management for three months in SARS-CoV-2-infected males who developed a fever [92].

## 7. SARS-CoV-2 and Male Hormonal Dysfunction

Male sex hormones secreted from the testes are crucial to the process of spermatogenesis. Their synthesis is regulated by the hypothalamus, pituitary, gonad (HPG) axis. Changes in levels of gonadotropins and testosterone in male COVID-19 patients might be caused by dysregulation of the HPG axis [93]. Schroeder et al., in a cohort study of 45 critically ill male COVID-19 patients, found 68.6% had low testosterone and 48.6% had low dihydrotestosterone levels [94]. Rastrelli et al. reported that lower total testosterone (TT) and calculated free testosterone (cFT) were found in the group of clinically deteriorating or deceased patients (*n* = 4) as compared to the group of patients with stable or improving clinical conditions (*n* = 27) [95]. 

A retrospective single-center study involving 119 COVID-19 patients and 273 age-matched control men reported that infected men had no statistically significant differences in serum testosterone and FSH levels compared with the controls. Yet they revealed a higher serum LH level and significantly decreased ratios of T/LH and FSH/LH. This study implies that SARS-CoV-2 tends to impair the function of Leydig cells [96]. In addition to Leydig cell dysfunction, there is increasing evidence that hypothalamic dysfunction is responsible for the impairment of male sex hormones secretion [97]. Moreover, the substantial ACE2 expression and local lesions recorded in the hypothalamus of COVID-19 patients increase the risk of HPG axis dysfunction [98,99].

Apart from the direct impact of SARS-CoV-2 infection on patients, the dysregulation of the HPG axis can be also induced by psychological factors. COVID-19 may trigger several mental problems, including anxiety, depression, post-traumatic stress disorder, and sleep disturbances, which negatively affect semen parameters [100,101]. 

It is necessary to further investigate the relationship between male sex hormones level and SARS-CoV-2 infection in prospective long-term studies to evaluate hypogonadism and sexual dysfunction caused by abnormal sex hormone levels.

## 8. COVID Vaccine and Fertility 

The COVID-19 vaccine acts by training the human organism to develop immunity and fight against the virus that causes COVID-19. This may prevent future infectious disease. The COVID-19 vaccines target the spike protein (S) of SARS-CoV-2 virus which induce in the human body the production of the neutralizing antibodies. mRNA vaccines are characterized by high potency connected with an adjuvant. These types of vaccine induce activation of two complementary parts of immunity: B cell responses by antibody production and T cell cytotoxicity as cellular response. The main purpose of vaccination is to induce herd immunity, which should contribute to elimination or significant decline in disease transmission in a population. Researchers estimated that 67% of the population is required to be vaccinated for herd immunity to SARS-CoV-2 [102]. The mRNA (Pfizer-BioNTech and Moderna) and viral vector (AstraZeneca) COVID-19 vaccines are novel in design and, to date, are the first mRNA and viral vector vaccine trials to have been comprehensively evaluated for disease prevention in people. The latest publication of the Association of Reproductive and Clinical Scientists and the British Fertility Society indicates that is no evidence that any forms of COVID-19 vaccination can affect male or female fertility [103]. In the ongoing research, there are no data about the COVID-19 vaccines influencing infertility. It is also hard to find in literature any credible scientific theories explaining the mechanism of influence of COVID-19 on female infertility. 

Researches from Charles River Laboratories France Safety Assessment SAS prepared animal experiment using COVID-19 vaccines; during the experiment, female rats were administered BNT162b2 (30 μg mRNA/dose) intramuscularly 21 and 14 days prior to the start of mating and on gestation days 9 and 20 [104]. BNT162b2 is a lipid nanoparticle (LNP) formulated nucleoside-modified mRNA encoding SARS-CoV-2 spike protein. This vaccine preparation was authorized for emergency use in the United States and conditional approval in Europe. It demonstrated 95% efficacy in a clinical trial and over 90% effectiveness in preventing COVID-19 in patients aged 16 and older [105]. According to data achieved in the experiment, no BNT162b2-related effects on female fertility were observed. The presence of neutralizing antibodies was confirmed in dams, fetuses, and offspring. All examined parameters and behaviors, including estrous cycle, pre-coital interval, mating, fertility, and pregnancy indices were lacking any departure from normal as compared to the control group. These results are very promising and confirm early assumptions about COVID-19 vaccines. In a short amount of time, after vaccination against SARS-CoV-2 some side effects may occur—injection site pain, fatigue, fever, swelling, muscle pain, headache, cough, disturbed appetite, diarrhea, and nausea [106].

## 9. Conclusions

According to the currently available literature, it seems reasonable to assume that SARS-CoV-2 infection might affect both female and male reproductive organs, and to some extent could also affect human fertility and the pregnancy course and outcome. The latter is highly possible due to the reported possibility of vertical transmission of the virus. Based on the studies’ results, the elements of both male (testes, semen) and female (ovaries, uterus) reproductive organs might be the potential target of SARS-CoV-2 infection. Even though there are reports indicating the possible role of the viral infection on the reproductive organs, very little is known about the long-term effects of the infection. Therefore, it is of major importance to provide in-depth research explaining the underlying mechanism of SARS-CoV-2 infection and its impact on human reproductive organs and fertility.

## Abbreviations

ACE—Angiotensin-Converting Enzyme; BMI—Body Mass Index; BSG—Receptor Basigin; BW—Birth Weight; cFT—Calculated Free Testosterone; CTSL—Cysteine Protease Cathepsin; HPG—Hypothalamus, Pituitary, Gonad; IVF—In Vitro Fertilization; LNP—Lipid Nanoparticle; MVM—Maternal Vascular Malperfusion; PCR—Polymerase Chain Reaction; PMS—Premenstrual Symptoms; RT-PCR—Real Time Polymerase Chain Reaction; SARS-CoV-2—Severe Acute Respiratory Syndrome 2; TEM—Transmission Electron Microscope; TMPRSS2—Transmembrane Serine Protease 2; TT—Total Testosterone; WHO—World Health Organization.
